# Personal attributes and competencies required by community health workers for a role in integrated mental health care for perinatal depression: voices of primary health care stakeholders from Surabaya, Indonesia

**DOI:** 10.1186/s13033-018-0224-0

**Published:** 2018-08-14

**Authors:** Endang R. Surjaningrum, Anthony F. Jorm, Harry Minas, Ritsuko Kakuma

**Affiliations:** 1 Centre for Mental Health, Melbourne School of Population and Global Health, The University of Melbourne, Melbourne, Australia; 2 Faculty of Psychology, University Airlangga, Surabaya, Indonesia

**Keywords:** Community health workers, Characteristics and competencies, Perinatal depression, Primary health care, Indonesia, Human resource for mental health

## Abstract

**Background:**

Non-professional community health workers have been widely reported as possibly having a role in mental health. In Indonesia, their role is currently being introduced in the national health system for perinatal depression. Prior publications have shown that it is generally considered feasible and acceptable by key stakeholders for community health workers to identify and refer women experiencing mental health issues during their perinatal phase to primary care. However, characteristics and competencies required for these workers have not yet been identified.

**Methods:**

62 participants from four groups of stakeholders in primary health care in Surabaya were interviewed, including program managers, health workers, community health workers (CHWs), mental health specialists, and pregnant and postpartum women. Semi-structured questions were used to explore participants’ views about characteristics and competencies required by CHWs to identify and refer perinatal depression.

**Results:**

Literacy and social skills were seen as basic characteristics required for CHWs to contribute to perinatal identification, together with willingness to volunteer and time availability. Participants identified females in the age range 30–50 years who have experienced pregnancy as being preferable. To ensure competency, training addressing knowledge about maternal life and depression, and communication skills are regarded as prerequisites for the role.

**Conclusions:**

The results are consistent with WHO guidelines for informal workers working with people with mental disorders in non-specialised settings. The results provide a rationale for the criteria to be met when informal workers are to be involved in primary care mental health area and provide information for the development of training in the identification of perinatal depression.

## Background

Community health workers (CHWs) play an important role in health care in Indonesia. CHWs provide informal community care and are recruited based on willingness, ability, and availability to run a community-based health service voluntarily. To equip CHWs with skills in health care and ensure capability, they are trained in health-related areas, mainly in maternal and child health [1], and in other health areas as required. CHWs are also increasingly becoming important in mental health care. For instance, CHWs are currently expected to be part of a national community mental health action team at the primary health care level [2], and to play a role in identification of depression among pregnant and postpartum women [3, 4]. Despite the lack of national record on perinatal depression, study in Surabaya reported prevalence of perinatal depression was quite high at 22% [5], calling to involve CHWs for identification of perinatal depression.

Community health workers have a role in mental health care in many developing countries [6] as an approach to alleviating shortages in the mental health workforce [7]. Depending on local needs, CHWs are trained to contribute to a range of tasks such as detection, diagnosis, treatment, and prevention of mental disorders [7–9]. Studies in India, Pakistan and South Africa have shown that CHWs have effectively filled roles as counsellors and psycho-educators using complex treatment [10–13], including screening and providing interventions to patients with common mental disorders [10]. Intervention studies in Pakistan and South Africa have found that CHWs can be trained to identify and provide intervention for maternal depression [13–15]. CHWs in these studies completed secondary school and were trained in communication skills, psychoeducation on perinatal depression, and intervention technique using stress/anger management [14] and/or cognitive behaviour therapy [16]. However, very few studies have investigated the personal characteristics and competencies required for CHWs to carry out these tasks [17, 18] despite acknowledgement that knowledge to specific mental health issues and communication skills is important in improving competencies to deliver support.

The WHO defines competence as “…a level of performance demonstrating the effective application of knowledge, skill and management” [19, p. 33]. Competencies reflect three elements: (1) knowledge, understanding and judgment; (2) cognitive, technical and interpersonal skills; and (3) a range of personal attributes and attitudes. This competencies framework suggests three areas of competency for informal CHWs: (1) basic understanding of mental disorders, including understanding symptoms of mental disorders; (2) basic counselling competencies, including listening and communication skills, especially empathic listening; and (3) advocacy. Communication is the most frequently suggested skill for workers dealing with mental health-related issues [16, 18]. The importance of communication skills is demonstrated in the World Health Organization (WHO) Mental Health Gap Action Programme Intervention Guide (mhGAP-IG) on non-specialized health settings, which offers guidelines for mental health actions in developing countries. This document suggests effective communication skills as a general principle of essential care, along with respect and dignity. Description of these skills include preparing an environment that facilitates open communication; being friendly, respectful and non-judgmental; using good verbal communication skills; and being sensitive towards difficult experiences [20].

In a recent qualitative study in Indonesia investigating feasibility of mobilizing CHWs to contribute to identification and referral of women with perinatal depression, it was found that not all CHWs currently working in maternal and child health care are perceived capable of carrying out this task [21]. The findings highlighted clear need to define CHWs characteristics and competencies in order to recruit and train CHWs who may contribute to perinatal mental health care. The aim of this qualitative study was to explore the characteristics and competencies required for CHWs to effectively identify and refer women with perinatal depression in Indonesia by examining perceptions of key stakeholders.

## Methods

### Research setting and participants

This article reports on part of a broader study conducted in Surabaya, Indonesia, to investigate feasibility and acceptability of mobilising CHWs for perinatal mental health care. Four stakeholder groups in primary health care (PHC) participated: (1) program managers from the district health office and primary health care services (integrated health service posts) in villages, (2) health workers, i.e. primary care doctors (GPs), nurses, midwives and counsellors in PHC centres, and CHWs from three villages, (3) mental health specialists, and (4) service users, i.e. pregnant and up to 1 year postpartum women. These groups were identified based on PHC system at district level [21, 22].

Program managers were selected from the district health office and three villages. Health workers were recruited from each of the three PHC centres at sub-district level recommended by the health office. Within the catchment area of one of three PHC centres, three villages were randomly selected to recruit community program managers. From each of these villages, two community-based integrated health service posts were identified from which CHWs and service users were recruited. Mental health specialists were recruited from one district hospital. Further details regarding participants are described in Surjaningrum et al. [21].

### Data collection

Participant views on CHW required competencies were collected through face-to-face semi-structured interviews. Participants were interviewed about their perceptions on attributes and competencies for CHWs involved in identification of perinatal depression, and more specifically about personality, educational background, gender, experience, and other factors suggested by participants. Views on training needs of CHWs were also gathered, including whether they could be trained and content of training required. The first author (ES) carried out interviews in Indonesian with all participants. Interviews were audio-recorded with participant permission and subsequently transcribed.

### Data analysis

Interview data (interview transcriptions) were managed using NVivo 11. Data analysis was conducted using framework analysis [23, 24]. Elements of competencies as defined by WHO [25], were used as a coding framework which covered knowledge, a range of skills (cognitive, technical, and interpersonal), and a range of personal attributes (age, gender, education, and personality). Data analysis was conducted in Indonesian to prevent potential loss of meaning through translation. Transcriptions were translated into English when the analysis process moved to mapping and interpretation. This approach was applied to ensure interpretation was credible by allowing English-speaking collaborators to check and discuss the data.

### Ethics and institutional approval

Ethics approval was obtained from the University of Melbourne on May 20th, 2015 (Ethics No. 1543833). Approval to conduct the research in Surabaya was granted by the Department of Health, Surabaya.

## Results

### Participants

The data were collected between June and August 2015. 62 participants were interviewed. Almost half (n = 28) were service users. All CHWs and community program managers were female, Javanese, above 40 years, and had completed high school. Other stakeholder groups were more diverse in their characteristics, as presented in Table 1.

**Table 1:** Socio-demographic summary of participants

Characteristics	Stakeholder groups					
	Health workers (n = 12)	Community health workers (n = 12)	Mental health specialists (n = 5)	Program managers (n = 5)	Service users (n = 28)	Total (n = 62)
Gender						
Female	11	12	2	5	28	58
Male	1		3	0		4
Age group						
15–19					3	3
20–24					4	4
25–29	3		1		10	14
30–34	2				7	9
35–39	4		1		4	5
≥ 40	2	12	2	3		19
Unknown	1		1	2		4
Ethnicity						
Javanese	9	12	4	5	23	53
Madurese	2		1		4	7
Other	1				1	2
Level of education						
< High school					5	5
High school		12		2	19	33
> High school	12		5	3	4	24
Employment						
Employed	12		5	2	5	24
Self-employed					6	6
Unemployed		12		3	17	32

### Characteristics and competencies

Competencies required for CHWs to carry out perinatal depression identification include knowledge required, skills, personal qualities, and personal attributes. Participants advised a range of skills (cognitive, technical, and interpersonal), and a range of personal attributes such as age, gender, education, personality, motivation, and experience. Table 2 presents summary of the most frequent topics identified through the semi-structured interviews.

**Table 2:** Summary of CHWs' competencies and attributes

Competencies	Knowledge, skills, attributes	Qualities and specific topics
Knowledge and understanding	Knowledge (about)	Pregnancy: physical and psychological changes during pregnancy
		Maternal life
		Children development
		Perinatal depression: symptoms of depression and strategies of detection
	Attitude	Having positive attitude towards the mental health of pregnant women
		Being confident towards CHWs’ role for future generation
Skills and personal qualities	Communication skills	Having good communication techniques, i.e. manner of speaking/asking questions and using nonjudgmental and simple language
		Being aware to the confidentiality of an issue
	Social skills	Socially active and able to socialize well
		Being alert to community needs
		Respect for cultural diversity
	Personality	Open-minded and have problem-solving skills
		Mature, patient, and caring to others
Personal attributes	Age	Being 30–50 years old is the most preferable; below 30 is less trusted and lack of skills; above 60 is less productive
	Level of education	Having basic literacy (reading and writing) is necessary, but having a minimum of high school educational background is more preferred
	Sex	Female is preferred, but male is acceptable
	Motivation	Self-willingness and are not money-driven, *‘ikhlas’*
	Experience	Have experience in marriage, pregnancy, and working with mothers

#### Knowledge and understanding

Participants shared their perspectives on the types of knowledge or understandings required by CHWs for identifying perinatal depression. In general, CHWs require basic knowledge about pregnancy, maternal life, child development, perinatal depression, communication techniques, and specific knowledge, such as how to change attitudes, and risk factors.

Knowledge about pregnancy and physical and psychological development during pregnancy was recommended by service providers (e.g. midwives and CHWs), mental health specialists, and service users. These groups stated that CHWs should be informed about the physical and hormonal changes of pregnant women that may result in psychological changes. These changes may affect health of mothers and their relationship with the infant and other people in their life. Some women or mothers in a good psychological state may adjust to changes and are more likely to take care of themselves during pregnancy, but other women may need help. CHWs who understand that pregnancy is not always associated with happiness may have better ways of approaching mothers. A specialist stated:

*“People think that pregnancy is a happiness event, however not all people are happy when they are pregnant. …When a CHW meets pregnant women and finds changes, she needs to know [whether a woman needs help]” (Sp 4)*

Another important area of knowledge is behavioural signs of mothers and/or their children that indicate depression. Health workers suggested that presentation of depression during pregnancy may be different to that after giving birth. Therefore understanding the signs and symptoms is necessary. CHWs and service users suggested CHWs should understand detection methods, including how to use a screening tool.

A specialist suggested that CHWs need to have positive attitudes towards the mental health of pregnant women. This specialist stated that it is important to build a paradigm and understanding that what CHWs are doing is important as an investment in the future generation.

#### Skills and personal qualities

##### Communication skills

Participants emphasized the importance of personal qualities and skills. For those who work with mental, neurological and substance use disorders in non-specialized settings, WHO suggests effective communication skills and promoting respect and dignity are general principles [20]. These principles were also reported as necessary skills by study participants from all stakeholder groups.

Knowledge about communication techniques is necessary. Health providers and specialists discussed a need for communication techniques that do not offend mothers, including verbal techniques, such as way of talking, tone, and language used, and non-verbal methods, such as body language, and interview techniques. Mothers recommended that information be provided to CHWs on strategies regarding asking questions that reduce the risk of mothers/women feeling scared or reluctant when gathering information from people less involved in the rest of community (e.g. migrant workers), and when suggesting solutions.

Service users and CHWs listed in detail communication skills they expected, such as manner of communicating with others, talking softly, rather than being reactive or directive, to create comfort, not using medical terminology, and having a non-judgmental attitude. CHWs perceived willingness to listen to others as an important skill in communication:

*“Those who will to listen, people who can talk smartly, finding opportunities among words. Yes, I think those things” (CHW J4)*

Some participants highlighted the importance of people who can maintain confidentiality. In contrast, a communication style that is directive, straightforward, involving a superior tone, and less empathetic, referred to in a local terms as ‘*judes*’ (salty), is undesirable.

However, a mother stated that CHWs do not need to improve communication skills because, -as village people living in the same area as users, they know how to talk to neighbours. Indeed, a CHW explained that a person is assigned as a CHW because she is articulate, and able to persuade and educate others. Thus, CHWs have good communication skills with people in the village:

*“[the centre] selects CHWs: this person is smart and able to persuade others, is articulate, can provide counselling and broader perspectives. Then that person would be selected to be a CHW and [get trainings]” (CHW NS3)*

##### Social skills

All stakeholder groups stated that CHWs in this role should have social competencies. These skills refer to the ability to socialise well with others and to participate in social activities. Being a CHW for some time is regarded as preferable, even though new CHWs receive training before carrying out tasks, as being used to dealing with the community is helpful when encountering women with emotional problems such as depression. In addition, CHWs are expected to have sensitivity to, and awareness of, community needs, and those who have already participated in the CHW role are thought to be more likely to have this quality:

*“CHWs work on several tasks, but for this task I recommend those who are active and have concerns [about issues in the community]” (Nr 3)*

##### Personality and problem-solving skills

Several personal qualities are expected of CHWs, such as being broad-minded and having problem-solving skills. Specialists proposed problem-solving skills as a competency that enables CHWs to provide feedback or offer solutions to users. In addition, perinatal depression may be caused by social and cultural factors. Therefore taking care of such women requires CHWs with open views about social and cultural life. This quality is associated with level of education. A mother stated:

*“If she [a CHW] has higher education she would have broader experience, is accurate, and can offer solutions because she has learnt more. Those [CHWs] who are less educated but have more experience may also [provide solutions], just like most CHWs in our community” (Pr C1)*

Dealing with women in different emotional situations required patience, attentiveness, and care. These needs were expressed by a pregnant woman who perceived the necessity of being patient and talking softly to women:

*“…be patient, able to offer solutions, talk in good manner and soft, because she will talk to people who may have problems…” (Pr E4)*

For service providers, it is necessary to have CHWs who are loyal and committed to assist the community under supervision of the PHC centre.

#### Personal attributes

##### Age

**Fig. 1:**
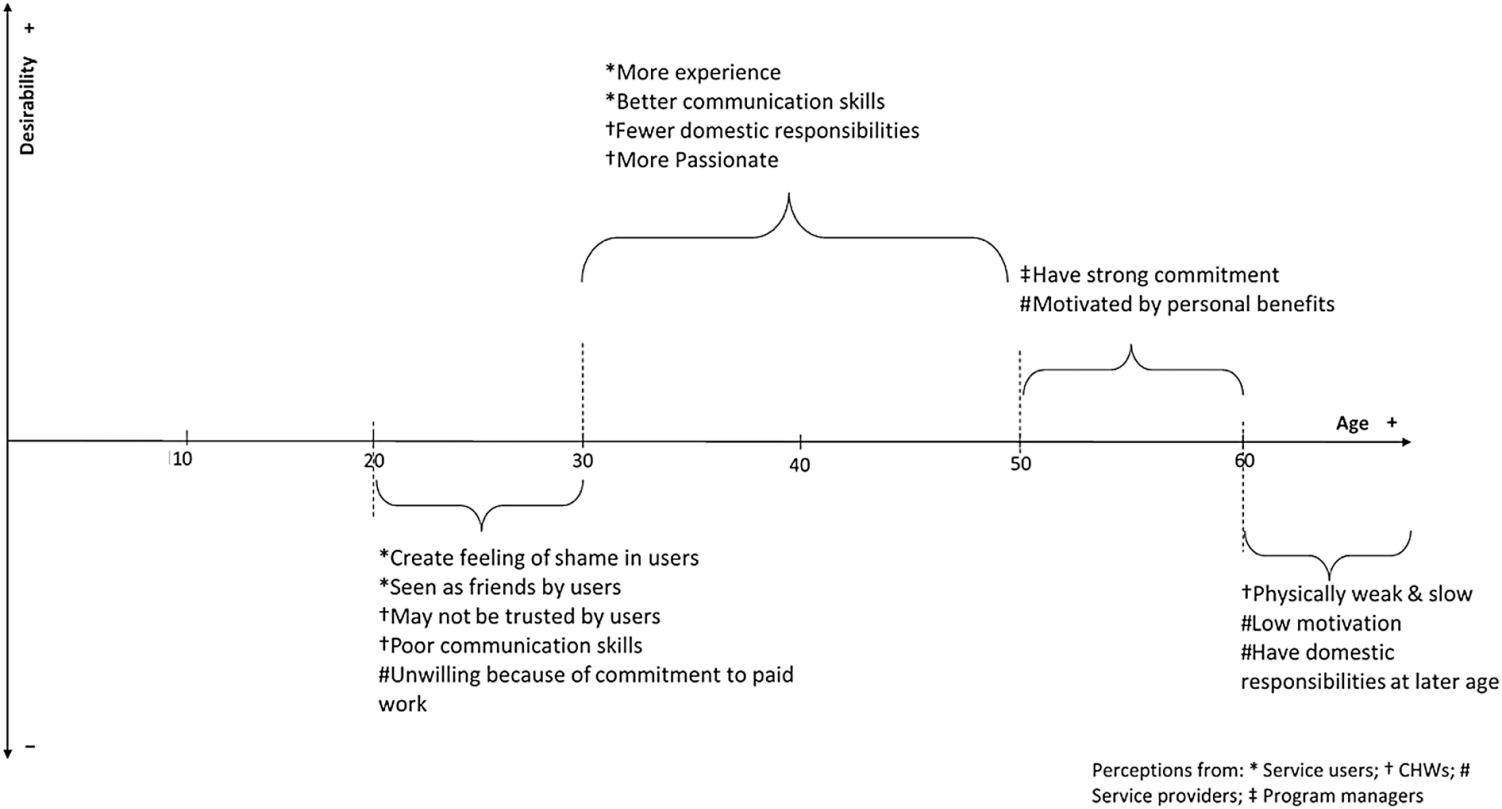
Desirable characteristic and the reasons for age preference

Figure 1 summarizes the desirable age range and reasons for age range preference as expressed by participants.

As described by research participants, the desirable age of CHWs to participate in this role is between the 30s and 50s years of age (Fig. 1), with the most preferable age range being the 30s to 40s. However, service users and CHWs perceived that personal characteristics, availability, and competencies are more important than a person’s age and that the preferred age range correlates with certain competencies. For example, an older CHW is expected to have experience of pregnancy and better communication skills, which is less likely in someone in her 30s. Similar expectations applied to other characteristics, such as responsiveness to user’ needs, commitment to tasks, and maturity. A mother stated:

*“The [existing] CHWs are already old. I reckon it is better to choose those in their 30s* *because they are more experienced. If they are below 30, they cannot communicate well and cannot approach well to the community” (CHW M1)*

In addition, people in their 30s and 40s were considered to have fewer domestic responsibilities. Most women in Indonesia marry and have children in their 20s and therefore have less domestic tasks in their 30s and 40s.

Service providers had a preference for CHWs in their 50s as they perceived people in this age group are more motivated by personal benefits gained from taking on this role, e.g. gaining knowledge about perinatal mental health that can be transferred to their own family.

People below 30 and above 60 years of age were less preferred to be a CHW involved in the task of depression identification, for reasons that varied among groups of stakeholders. Service users referred to personal feelings that influence age preference. For example, they preferred not to see CHWs below age 30 or of the same age as themselves, because it may generate feelings of shame from sharing psychological experiences and because the relationship would seem “*just like talking to one’s own friends*”. In agreement with service users, CHWs stated that this age group is less likely to be respected and trusted, and consequently service users may not listen to them. In addition, young people are busy with paid employment and may not be available to take on the role.

On the other hand, people over 60 years of age were seen as too old, less productive, and reluctant to connect to the PHC centre, with many having responsibility for taking care of grandchildren. Persons of this age were also seen as sometimes having bad communication skills because they may talk in a directive way and with a lack of empathy. One participant stated:

*“If they are too old, they are reluctant to come to PHC centre, sometimes they also have responsibility such as taking care grandchildren. Therefore, it is better neither too young nor too old…at about 30 to 50* *years old. That’s the range” (Cs 3)*

##### Education

Many participants stated that level of education is not a principal requirement for CHWs to undertake a depression identification role. For CHWs, basic competence in reading and writing is sufficient as CHWs receive training in related services. Participants also perceived that some people may have a low level of education because of financial constraints rather than inability to learn, and that they may have other relevant qualities, such as social skills and work skills. Specialists were concerned about difficulty finding people for this task if a higher level of education were a requirement:

*“…if we set a specific level of education, we will face problem in finding the CHWs. So we focus on people who are active and able to work together. To be honest, it will be better if we consider education because understanding about depression requires higher knowledge which is identical to higher level of education. However, practically we won’t get them” (Sp 1)*

Instead of education as a CHW criterion, program managers perceived willingness as the important requirement for a CHW once a basic literacy level was met:

*“Usually for CHWs we do not see their education level, because the principle is she will to take the tasks. If we consider their education, then no one may to join” (CPM 3)*

Service users stated that being smart is not always related to educational background. This group emphasized experience, willingness, and personal characteristics as essential criteria. A person who is attentive and can make others feel comfortable to talk to was seen as more important than having a particular educational level. A woman said:

*“If someone has a bachelor degree but she is not willing to be a CHW, cannot get along with others, talks to others in bad way, then I will not feel comfortable talking to her. So, the more important thing is personal characteristics of the person, not the level of education” (Ppt A5)*

Although many participants considered literacy is more important than a certain level of education, most participants recommended people with either a junior or senior high school background as sufficient if a specific level of education were to be required. This level of education is associated with acceptable communication skills, and people with this background are not difficult to find in the community. CHWs with a primary school background are less preferred, as their communication skills are considered poorer.

Some specialists and service users stated that a tertiary level education would be better for the CHW role, because CHWs with a degree are more open-minded, have better knowledge in general and about depression specifically, and have better problem-solving skills. However, finding people to perform the role of a CHWS with this educational level is not easy and, even if available, they are not always able to communicate and socialize in an appropriate manner with service users.

##### Sex/gender

A majority of participants suggested that women should carry out the CHW role. Most service users stated women promote comfort and less shameful feelings for service users, and generally have gender-based sensitivity regarding women’s feelings and experience in pregnancy and caring for infants. Women are also likely to have more time to commit to the role, as most men have work obligations. Service users raised concerns regarding relationship issues that may occur if CHWs were men, e.g. male CHWs may aggravate marital problems of service users. A program manager preferred women simply because of the reality that most CHWs in her area are women. A pregnant woman said:

*“I prefer to have women because they have experience being pregnant. If they are men I will be reluctant and hesitate, even with our own husbands sometimes we do not disclose… Men cannot feel what women do, how pregnancy feels like. Men can talk only” (Pr C5)*

##### Motivation

Service providers and community program managers focused more on willingness to take on the CHW role as an important criterion. Motivation as a criterion aligns with general requirements stated in the national guideline on management of ISPs [1]. Willingness means that the person wants to carry out the task voluntarily and not be driven by financial considerations. Rather than tangible benefits, some CHWs were motivated by an Islamic-related value called ‘*ikhlas*’, a belief that people receive intangible reward indirectly from God. This term is common among CHWs as it is a motivation to remain in this voluntary role without expecting a salary:

*a CHW has to be ready to social work, has to have KMS [card to go to heaven],* ikhlas*…” (CHW NS3)*

##### Experience

In addition to competencies, stakeholders highlighted the importance of experience. Several forms of experience were proposed by different stakeholder groups, e.g. service users preferred people who have had experience of marriage and pregnancy:

*“Certainly those who already have experience in giving birth, being pregnant, having children. Because experience is important, so before helping others she has experienced those situations. At least we can share” (Ppt E4)*

Experience as a CHW who has worked with women and mothers is strongly recommended by service users. This suggests that recruitment of CHWs for depression identification should be from among existing CHWs. A mother said:

*“New persons have less experience. It’s better if the existing ones are recruited and then be trained, [because] they already have experience. While the new ones need to be guided” (Ppt A4)*

## Discussion

This study investigated characteristics and competencies that CHWs require to participate in integrated mental healthcare for perinatal depression identification, including knowledge, skills and personal qualities, and personal attributes or characteristics. Findings show CHWs require knowledge about pregnancy and maternal life, child development, and perinatal depression. Knowledge about depression is expected to assist CHWs to identify signs and symptoms of depression. It is also suggested to train CHWs to use a screening tool for perinatal depression. The most recommended skills for CHWs in this task are communication and social skills. Other characteristics required for these CHWs are motivation, such as willingness and availability, basic literacy, and women who have experienced pregnancy and are in a certain age range. These findings add information from previous studies and available guidelines about CHWs working in general care and specifically for maternal mental health care. The findings could also become a foundation to develop particular guidelines for recruitment and training of the workforce.

The finding that CHWs require knowledge about pregnancy and maternal life, child development, and perinatal depression is consistent with previous studies where CHWs were provided psycho-education about pregnancy, child development and maternal depression in order to identify and treat mothers with depression [13–15, 18]. Studies on the role of CHWs in mental health care also reported that CHWs were able to identify a certain mental disorder being educated [10, 11, 26, 27], reinforcing the role of specific mental health-related knowledge as competencies required for the role of CHWs in mental health identification. Knowledge regarding perinatal depression and anxiety are within training modules addressing maternal depression, i.e. the Thinking Healthy Program, a manual for management of perinatal depression published by WHO [16]. Consistency with previous studies and international guidelines underscores the knowledge element of competencies for CHWs to carry out perinatal depression identification.

The findings that communication, social, and problem solving skills are expected for the CHW role by most stakeholders have been reported in previous studies [8, 16, 28]. Several studies emphasize communication as a basic skill that is required by CHWs working in the mental health area in diverse contexts. This skill is a core competency for human resources in the mental health area [25]. More specific behaviors reported in this study, such as attentiveness and empathy, listening, and asking questions, are also listed as among behaviors and attitudes within communication skills recommended by mhGAP-IG [20]. In addition, social skills are important due to CHW involvement with the community they serve. These skills were perceived by participants as understanding of social context, being socially active, and/or being aware of community needs. Having these skills means that CHWs have a strong bond with, and social–cultural understanding of, the community. Involvement in the community through social activities is part of ‘previous experience’ required in CHW recruitment [29], where CHWs should be chosen from and by the community they serve [30]. In this study context, internal recruitment of CHWs in maternal mental health means they should be selected from existing CHWs as their on-going community participation indicates their social skills and sensitivity to, and awareness of, community needs, indicating them asmore appropriate in the role of identifying mental illnesses in the community [31]. For Surabaya, CHWs^MCH^ (CHWs working on maternal and child health whom the study addresses) who are known by pregnant and postpartum mothers are the most suitable ones for depression identification. More specifically is from CHWs^pregnancy^, who are mostly CHWs^MCH^ assigned to regularly monitor the health and pregnancy risk of pregnant and postpartum mothers [21]. Appointing the role to CHWs^pregnancy^ means the new task accompanies existing tasks and therefore will not be an extra burden and could increase productivity [32, 33]. However, this possible issue may not the case in this context from the catchment area and work environment point of view [33]. With the catchment area, a CHW in Surabaya covers 50 households [21], however pregnant mothers made up only about 4% [34] leaving them to provide services to 2–4 pregnant and postpartum women within walking distance area. Other elements that could be managed is organisation of tasks and supervision. It is reported that mental health assessment for pregnant and postpartum mothers was commonly performed per trimester of pregnancy and at certain time postnatally [35–37] which can be done at a single client visit during regular health status monitoring by CHWs^pregnancy^. Moreover, the availability of a counsellor (psychologist) in every health centre enables an arrangement of supervision from qualified professionals within a monthly refresher program [21]. Considering to use checklist as an aid job which that was raised by health workers in implementing the programme could also overcome this potential issue.

Findings of this study about CHW attributes/characteristics are consistent with characteristics required for CHWs in general, and in the mental health area. However, attributes such as gender, age, education and experience are very much associated with women’s health. Findings on the preference for female CHWs to carry out this role is very specific in the context of maternal depression as a women’s health issues. This is also evident in early development of the role of CHWs in maternal and child health where women were most preferred [30, 38]. The same argument also applies to the requirement for CHWs to have had experience in pregnancy, mainly by service users, as also highlighted in studies about CHWs working in different health areas [30]. On the other hand, characteristics such as willingness, ability, and availability are widely reported as general criteria in recruiting CHWs [30]. Indonesian guidelines about integrated health service posts, which CHWs work within, requires these three elements as basic criteria in selecting CHWs [1].

All CHW required competencies build the trustworthiness of CHWs in carrying out a role in maternal mental health. Knowledge about mental health of mothers and brief intervention or problem solving skills enhances ability of CHWs to identify women with depression and take necessary action, such as referral to relevant services. This action requires motivation or willingness to help others [33]. To do so, being aware of others’ feelings and building trust with service users, health services, and the community are necessary and may be associated with maturity that is related to age and level of education. This argument suggests the need for a higher level of education rather than basic literacy, with a previous report [30].

**Fig. 2:**
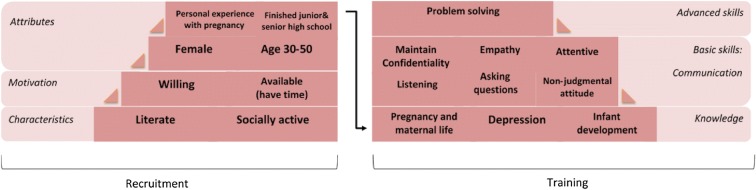
Characteristics and competencies required for recruitment and training needs

Findings of this study have implications for preparing CHWs to undertake a role in perinatal depression identification as part of integrated antenatal care [3, 4]. Guidance on selection and a training curriculum for CHWs in this role would add to general requirements of CHWs working in community-based health service posts [1]. Figure 2 presents a summary of findings arranged hierarchically, starting from general requirements and moving to more specific and advanced requirements that can only be acquired by CHWs through additional training. The characteristics, motivation, and attributes provide guidance on recruitment (left side), while the remainder (right side) provide guidance on development of a training curriculum. In addition, study findings fill gaps in information about competencies required for CHWs in health services. Recruitment criteria for health-related roles, such as level of education, preexisting experience and demographic characteristics related to place-based, i.e. that CHWs are from the same community as service users, were often presented without enough information about the reasons for determining the criteria [29].

There is a need for further exploration about the level of knowledge that can be transferred to CHWs, the depth of communication skills that CHWs can attain through training, and training methods that fit with their capability. CHWs are a good source of information in development of a training curriculum, as they have good understanding of cultural and interpersonal relations with the community, including acceptable communication and manners in approaching users.

## Limitation

Participants of the study were stakeholders of primary health care centres with limited networks at the village level. Management of the recruitment of CHWs is held by the Family Welfare Movement at the village level, whereas policy about management of recruitment and deployment is determined by the Family Welfare Movement at the district level. Therefore, perceptions from this organisation at district level are required to build better conclusions. In addition, the “recently established category” of CHWs^pregnancy^ were not invited to participate by the researcher since its existence was only recognised during the research process. Inclusion of their views would contribute to a better understanding of their role and responsibilities in future maternal mental health care and would provide better understanding about the characteristics and competencies required. The small sample size within health care system in the study area is a weakness, yet the number is big enough for a qualitative study. The use of unstandardized interview tool is among methodological limitation. Moreover, the district health system of the study site is very specific within the decentralized governance system, therefore generalizability of the findings to national context is poor.

## Conclusion

This study provides detailed information about competencies required by CHWs carrying out perinatal depression identification and referral. Many studies examining the role for CHWs in the mental health area have not been based on community voices, despite a common agreement that CHWs are selected from and based on criteria set by the community they serve. This study fills this gap in knowledge and provides a rationale for the criteria for CHW recruitment [29] in the specific context of maternal mental health care in Surabaya, Indonesia. Study findings show consistency with criteria being used in selecting CHWs in many studies, and with guidelines on recruiting CHWs in general and CHWs in mental health specifically. Elements such as knowledge and skills are also consistent with those stated in international guidelines for informal workers working in mental health published by WHO. Thus, generalisation of these findings to management of CHWs carrying out the role for perinatal depression identification in other contexts may be possible.
